# The Werner Syndrome Protein Suppresses Telomeric Instability Caused by Chromium (VI) Induced DNA Replication Stress

**DOI:** 10.1371/journal.pone.0011152

**Published:** 2010-06-16

**Authors:** Fu-Jun Liu, Aaron Barchowsky, Patricia L. Opresko

**Affiliations:** Department of Environmental and Occupational Health, University of Pittsburgh Graduate School of Public Health, Pittsburgh, Pennsylvania, United States of America; Roswell Park Cancer Institute, United States of America

## Abstract

Telomeres protect the chromosome ends and consist of guanine-rich repeats coated by specialized proteins. Critically short telomeres are associated with disease, aging and cancer. Defects in telomere replication can lead to telomere loss, which can be prevented by telomerase-mediated telomere elongation or activities of the Werner syndrome helicase/exonuclease protein (WRN). Both telomerase and WRN attenuate cytotoxicity induced by the environmental carcinogen hexavalent chromium (Cr(VI)), which promotes replication stress and DNA polymerase arrest. However, it is not known whether Cr(VI)-induced replication stress impacts telomere integrity. Here we report that Cr(VI) exposure of human fibroblasts induced telomeric damage as indicated by phosphorylated H2AX (γH2AX) at telomeric foci. The induced γH2AX foci occurred in S-phase cells, which is indicative of replication fork stalling or collapse. Telomere fluorescence in situ hybridization (FISH) of metaphase chromosomes revealed that Cr(VI) exposure induced an increase in telomere loss and sister chromatid fusions that were rescued by telomerase activity. Human cells depleted for WRN protein exhibited a delayed reduction in telomeric and non-telomeric damage, indicated by γH2AX foci, during recovery from Cr(VI) exposure, consistent with WRN roles in repairing damaged replication forks. Telomere FISH of chromosome spreads revealed that WRN protects against Cr(VI)-induced telomere loss and downstream chromosome fusions, but does not prevent chromosome fusions that retain telomere sequence at the fusion point. Our studies indicate that environmentally induced replication stress leads to telomere loss and aberrations that are suppressed by telomerase-mediated telomere elongation or WRN functions in replication fork restoration.

## Introduction

Telomeres are highly specialized chromatin structures consisting of tandem repeats of the TTAGGG sequence bound and regulated by telomeric proteins (shelterin) and a plethora of accessory factors. Located at the ends of linear chromosomes, telomeres prevent the DNA damage response (DDR) and repair machineries from recognizing and processing the ends as double-strand breaks (DSBs) [1]. Extensive loss of telomeric DNA and proteins induce telomere dysfunction and activation of numerous DDR proteins at the telomeres including phosphorylated histone H2AX (γH2AX), resulting in telomere dysfunction-induced foci (TIFs) [2], [3]. Telomere dysfunction causes chromosomal instability, growth arrest (senescence) or cell death [1]. Telomerase is a ribonucleotide enzyme that lengthens eroded telomeres to maintain cellular proliferative capacity and genome integrity [4]. However, most human somatic cells lack sufficient telomerase activity to prevent telomere shortening that occurs with every replicative cycle [5]. Defects in telomere length homeostasis and telomerase activity are associated with numerous human diseases including progeroid syndromes, cancer, bone marrow failure, and pulmonary fibrosis [6].

Accumulating evidence indicates that replication fork stalling or collapse at telomeric ends can lead to telomere loss or aberrations. Telomeric instability associated with defects in telomere replication are induced by polymerase inhibitors and agents that stabilize DNA G-quadruplexes or by depletion of shelterin TRF2 or POT1 proteins [7], [8], [9]. Loss of the WRN helicase/exonuclease results in Werner syndrome (WS), which is characterized by features of premature aging and cancer predisposition [10]. Cellular data support roles for WRN in the processing of stalled replication forks, and the recovery from replication stress [11], [12], [13]. The premature senescence, genomic instability and stochastic telomere loss phenotypes of WS cells can be rescued by expressing either WRN protein or telomerase [14]. These data indicate that telomerase can compensate for WRN roles at telomeric ends. WRN has been implicated in telomere replication. WRN localizes to telomeres in S-phase telomerase deficient cells and interacts with shelterin proteins TRF2 and POT1 [14], [15], [16]. WRN defective cells exhibit increased telomere loss particularly on sister chromatids replicated from the G-rich telomere strand [14]. These studies indicate that shelterin proteins together with telomerase or accessory proteins, such as WRN, are required to prevent telomere abnormalities resulting from endogenous obstacles to telomeric replication. However, their importance in protection against telomere loss due to exogenous or environmental effectors of replication stress is not known.

The environmental metal hexavalent chromium (Cr(VI)) is an important source of DNA replication stress. The inhalation of Cr(VI) particles is strongly linked to respiratory cancers in the occupational setting [17], and short telomeres are associated with increased risk for lung cancer [18]. The expression of telomerase in human fibroblasts significantly reduces Cr(VI)-induced cellular toxicity and genomic instability, however, Cr(VI) exposure does not significantly alter mean telomere lengths [19], [20]. Whether Cr(VI) impacts the integrity of individual telomeres, or induces telomeric abnormalities is unknown. Cr(VI) reduces to Cr(III) in cells which reacts with DNA and produces a broad array of lesions [21]. Guanine runs are hotspots for Cr(VI) mediated base substitutions and deletions, Cr-DNA adducts and further oxidation of 8-oxo-guanine [22], [23]. Importantly, Cr(VI) treatment of DNA templates *in vitro* induces DNA polymerase arrest with the most potent arresting lesions at G runs [24], [25]. Consistent with this, Cr(VI) exposure induces replicative stress indicated by S-phase arrest and S-phase dependent DSBs [24], [26], [27] which likely result from replication fork collisions with blocking lesions. Defects in DNA repair proteins that maintain replication fork stability, including WRN, lead to Cr(VI) hypersensitivity [11], [12], [28], [29], [30]. These findings indicate that telomeric repeats may be particularly vulnerable to Cr(VI) induced lesions that stall replication. The consequence of environmentally induced replication stress on telomere integrity is largely under-investigated.

In this study we examined whether Cr(VI)- induced replication stress impacts telomere integrity at the molecular and chromosomal level. We further tested the hypothesis that WRN and telomerase protect against Cr(VI)-induced cytotoxicity and genomic instability, partly by preventing Cr(VI)-induced telomere defects. We found that Cr(VI) exposure in human cells induces telomeric damage as indicated by telomere dysfunction induced foci (TIFs), and telomeric abnormalities associated with defects in telomere replication including telomere loss on chromatids. The latter was attenuated by telomerase expression, and exacerbated by WRN depletion. Thus, we provide novel evidence that environmentally induced replicative stress can impact telomere integrity, and offer a mechanistic explanation for the increased sensitivity of WRN and telomerase deficient cells.

## Results

### Telomerase protects against Cr(VI)-induced cytotoxicity and telomere instability

**Figure 1:**
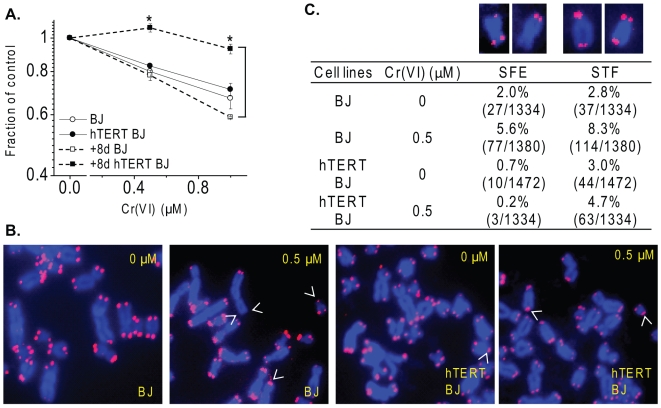
Telomerase expression attenuates Cr(VI)-induced cytotoxicity and telomere aberrations. (A) Cell viability assay. The cells were exposed to the indicated Cr(VI) concentrations for 48 h, and subpopulation of Cr(VI)-treated cells were subcultured in Cr(VI)-free medium for 8 days. (B) Images are representative telomere FISH of partial metaphases from cells exposed to 0 or 0.5 µM Cr(VI) for 48 h and cultured in Cr(VI)-free medium for 10 h. Arrows heads indicate telomeric defects that appeared in each of the focal planes of the Z-stack series (see Materials and Methods), although only one focal plane is shown. (C) Frequencies of Cr(VI)-induced telomere aberrations. Values in parenthesis indicate the number of abnormal chromosomes/total chromosomes. Telomere signal free ends, SFEs; sister chromatid telomere fusions, STFs. Insets show examples of two SFEs, followed by two STFs.

Previous studies showed that exposure to Cr(VI) significantly reduced survival of TERT- BJ cells compared to the TERT+ BJ cells [19], [20]. While Cr(VI) exposure did not alter mean telomere lengths in either cell line [19], the impact on telomere integrity was not examined. We predicted that telomerase may protect against Cr(VI) toxicity and genomic instability by preventing induced telomeric defects and dysfunction. First we confirmed the protective effect of telomerase against Cr(VI) toxicity by testing cell survival and replicative capacity of exposed cells [11]. After 48 h Cr(VI) exposure cellular sensitivity was not affected by telomerase status (Figure 1A), suggesting no significant difference in survival immediately following Cr(VI) exposure. However, TERT- cells demonstrated hypersensitivity at both 0.5 and 1 µM Cr(VI) compared to TERT+ cells (greater than 25% difference in survival) after 8 days of subculturing in Cr(VI)-free medium. Our data indicate that TERT- cells exhibited a significantly reduced capacity to recover and proliferate after Cr(VI) exposure, compared to TERT+ cells.

To determine whether Cr(VI) exposure can induce telomeric defects and whether telomerase can protect against such telomere instability, we examined individual telomeres in TERT- and TERT+ BJ cells after 0.5 µM Cr(VI) exposure for 48 h, followed by 10 h recovery. Telomeres on metaphase chromosome spreads were visualized by telomere fluorescent *in situ* hybridization (Telo-FISH) with a PNA probe (Figure 1B) [31]. Telomere defects manifested as telomere signal free ends (SFEs, lacking detectable telomere signal at one or more sister chromatids), sister telomere fusions (STFs), telomere doublets (the presence of more than one telomere signal at a chromatid end), and telomere-containing chromosome/chromatid fusions (T-CFs). Cr(VI) exposure increased the incidence of all types of the detectable telomeric defects more than two fold in TERT- BJ cells (Figure 1C). Importantly, telomerase expression dramatically reduced the incidence of SFEs by 28-fold after Cr(VI) exposure (5.6% of chromosomes in TERT- cells compared to 0.2% of chromosomes in TERT+ cells) (Figure 1C). Additionally, Cr(VI)-induced SFEs in TERT+ BJ was reduced 3.5-fold compared to untreated cells (0.7%). This strongly suggests the SFEs were due to telomere loss or truncation and not chromosome breaks in the sub-telomeric or genomic regions. While Cr(VI) induced a near 3-fold increase in SFEs in TERT- cells, compared to the untreated cells, no such induction occurred in TERT+ cells. Furthermore, nearly 2-fold reductions in Cr(VI)-induced STFs and T-CFs were observed in TERT+ cells compared to TERT- cells, but the induction of telomere doublets was not influenced by telomerase expression (Figure 1C, Table S1). In agreement with previous reports [19], we observed that 0.5 µM Cr(VI) induced chromatid breaks in TERT- cells (3/30 metaphases) (Table S1), but not in TERT+ cells. In summary we confirmed that telomerase protects against Cr(VI) cytotoxicity, and observed that telomerase expression dramatically reduced the incidence of Cr(VI) induced telomere signal free ends, consistent with protection against telomere loss.

### Cr(VI) induces telomere damage associated with cells in S-phase

**Figure 2:**
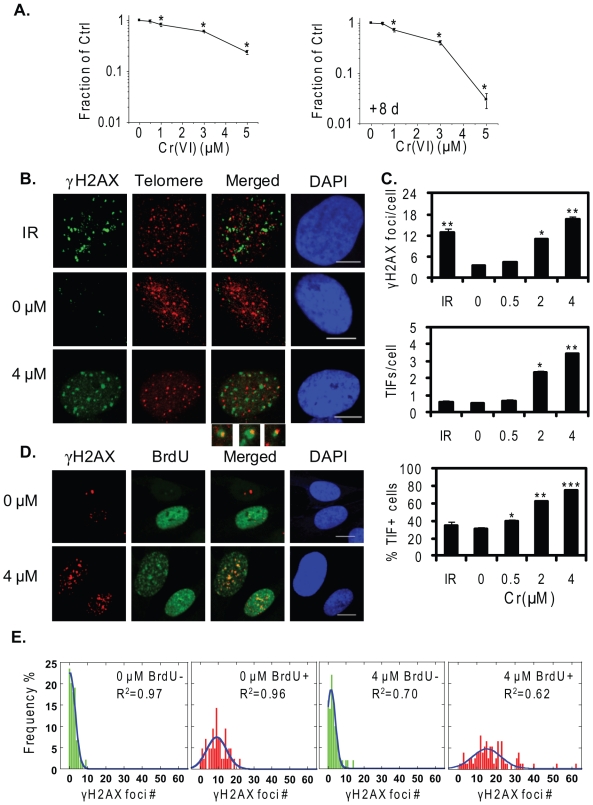
Cr(VI) induces replication-associated TIFs in lung fibroblasts. (A) Cellular toxicity of Cr(VI) upon 48 h exposure followed by no recovery or an 8-day recovery of a subpopulation of WI-38 cells in Cr(VI)-free medium. The symbol (*) indicates a significant difference from untreated in the indicated Cr(VI) concentration (p<0.05). (B) Confocal images of WI-38 cells exposed to the indicated Cr(VI) concentrations for 48 h. Cells were subjected to IF-FISH for analysis of γH2AX (green) and telomere (red) colocalization (yellow). IR: 1 Gy treatment, followed by IF-FISH after 1 h recovery. Bars, 10 µm. (C) Average γH2AX foci and TIF number per cell and the percent of TIF positive cells (% TIF+ cells). The % TIF + cells represent the percentage of cells containing at least one TIF foci. The data represent mean ±SE from two independent experiments, based on at least 50 randomly chosen cells for each Cr(VI) treatment. Bars with a different number of symbols are significantly different from each other (p<0.05). (D) Confocal images of γH2AX (red) association with S-phase cells, indicated with BrdU incorporation (green). Cells were exposed to 4 µM Cr(VI) for 48 h and then subjected to BrdU pulse labeling, followed by double immunostaining. (E) The frequency distribution of BrdU-negative and -positive cells is plotted against the number of γH2AX foci in the cells, based on a minimum of 120 randomly chosen cells from each Cr(VI) treatment.

Occupational Cr(VI) exposure poses a well established risk for developing lung cancer [21]. To investigate if Cr(VI) induces telomere instability in a relevant cell line for Cr(VI) carcinogenesis, we tested its effects in WI-38 lung fibroblasts. Interestingly, WI-38 cells were more resistant to low-level Cr(VI) exposure than skin BJ cells (Figures 1A and 2A). However, similar to BJ cells, the WI38 cells exhibited reduced survival and proliferation after an 8-day recovery from Cr(VI) exposure (Figure 2B). Based on the increased resistance of WI38 cells to Cr(VI), we examined their telomere damage with higher but still occupationally relevant Cr(VI) concentrations.

Telomere dysfunction-induced foci (TIFs) refer to the colocalization of DNA damage response factors with telomeres, and are widely used to investigate factors that induce telomere instability including compromised shelterin proteins or G4 stabilizing agents [2], [7]. Whether environmental genotoxins can induce TIFs is unknown. While we adopted the commonly used TIF term [2], in our studies these foci refer to telomere damage and not necessarily to un-repairable loss of telomere function. To test for TIF induction, WI-38 cells were exposed to Cr(VI) for 48 h and then subjected to IF-FISH to detect γH2AX colocalization with a telomeric peptide nucleic acid (PNA) probe. Both 2 and 4 µM Cr(VI) induced significant formation of γH2AX foci (more than 3 fold) and TIFs (more than 4 fold), and increased the percent of TIF positive cells (Figure 2C). In contrast, 1 Gy γ-irradiation, which induces random genome-wide DSBs, stimulated γH2AX formation comparable with 2 µM Cr(VI), but the TIF level was similar to the untreated group (Figure 2C). Our data indicate that while both γ-irradiation and Cr(VI) exposure induced γH2AX foci, only Cr(VI) induced a significant increase in DNA damage foci associated with telomeres.

Low levels of Cr(VI) exposure for 6–24 h induces replicative stress, indicated by γH2AX association with S phase cells [11], [26], [32]. Therefore, we predicted that the Cr(VI) induced TIFs occurred as a result of replicative stress at the telomeres. To test this we exposed WI38 cells to 4 µM Cr(VI) for 48 h, followed by pulse labeling with BrdU nucleotide analog, and determined γH2AX association with S phase cells by dual immunostaining (Figure 2D-E). BrdU negative cells displayed similar low levels of γH2AX foci number (peak near 1) in both the untreated and Cr(VI) treated cells. In contrast, the percent distribution in BrdU positive cells shifted towards the right, indicating increased γH2AX foci number with a peak at 9 for the untreated cells and a peak at 15 with a broader spread toward higher foci number after Cr(VI) treatment. Thus, Cr(VI) induced γH2AX foci in WI38 cells occurs in S-phase, consistent with results for other cell lines [11], [26]. Furthermore, the Cr(VI)-induced TIFs occurred in cells with higher numbers of γH2AX foci as observed in the BrdU positive cells. Of the Cr(VI) treated cells with >5 γH2AX foci, 93% (50/54) were TIF positive and 90% (69/77) were BrdU positive, indicating the TIF formation mechanism is most likely related to replication stress.

### Cr(VI) induces telomere defects in lung fibroblasts

**Figure 3:**
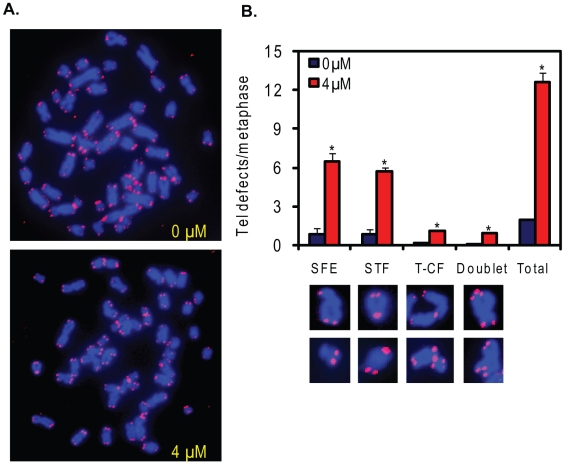
Cr(VI) induces telomere instability in lung fibroblasts. (A) Representative telomere FISH of full metaphases from cells exposed to the indicated Cr(VI) concentrations for 48 h and cultured in Cr(VI)-free medium for 10 h. (B) Cr(VI)-induced telomere instability. Approximately 60 metaphases from two independent experiments were analyzed to quantitate Cr(VI)-induced telomere defects. Bars with a symbol are significantly different from the untreated (p<0.05). The data represent mean ±SE from two independent experiments. Telomere signal free ends, SFEs; sister chromatid telomere fusions, STFs; chromosome/chromatid fusions with telomere signal at the fusion, T-CF.

To directly evaluate the consequence of Cr(VI)-induced telomere damage on telomere structure and integrity we stained individual telomeres on chromosome metaphase spreads using the Telo-FISH assay (Figure 3A). The total level of telomeric defects was significantly increased by 6.3 fold after 4 µM Cr(VI) exposure (Figure 3B). While all four types of telomeric defects, including SFEs, STFs, T-CFs and doublets were significantly increased (Figure 3B), the SFEs and STFs occurred at the highest frequencies. In both the untreated and Cr(VI)-treated group, most of the SFEs occurred at one chromatid end and the T-CFs were mainly chromatid fusions (data not shown). Although the levels of the doublets were lower than SFEs and STFs, the fold induction was higher (13-fold) compared to that of SFEs (7-fold) and STFs (7-fold). These data indicate that Cr(VI) exposure at low levels induces telomere instability in a relevant cell line for inhalation exposure.

### WRN protects against Cr(VI) cytotoxicity and localizes to telomeres upon Cr(VI) exposure

**Figure 4:**
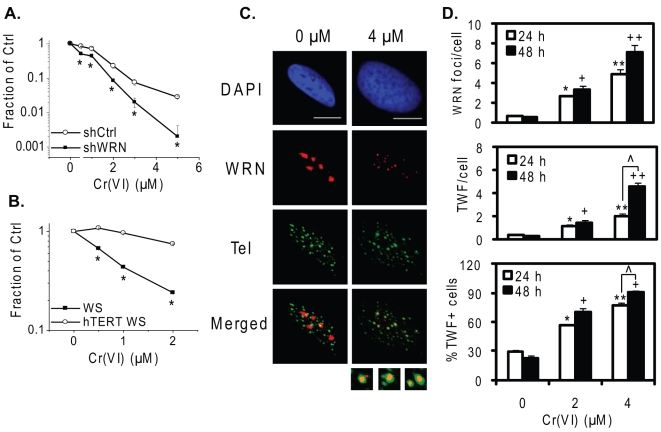
WRN protects against Cr(VI) cytotoxicity and localizes to Cr(VI) induced telomere damage. (A) Clonogenic assay of shCtrl and shWRN isogenic cells exposed to the indicated Cr(VI) concentrations for 24 h. (B) Cell viability assay. The cells were exposed to the indicated Cr(VI) concentrations for 48 h, and subpopulations of Cr(VI)-treated cells were subcultured in Cr(VI)-free medium for 8 days. In (A) and (B), the colony or cell number in the Cr(VI) treatments were expressed as a fraction of the untreated control. The data represent mean ±SE from at least three independent experiments. Bars with a symbol are significantly different from the untreated (p<0.05). (C) Confocal images of EYFP-WRN U2OS cells exposed to the indicated Cr(VI) concentrations for 48 h. Cells were subjected to IF-FISH for analysis of WRN (red) and telomere (green) colocalization (yellow). Bars, 10 µm. (D) Average WRN foci and telomere-colocalized WRN foci (TWF) per cell and the percent TWF positive cells. The data represent mean ±SE from two independent experiments, based on at least 50 randomly chosen cells for each Cr(VI) treatment. Bars with a different number of symbols are significantly different from each other (p<0.05).

Biochemical and cellular evidence support WRN roles in facilitating telomere replication, and in recovery from replication fork stalling [10], [33], [34]. Recently, we and others found that WRN protects against Cr(VI)-induced replicative stress [11], [12]. We reported that WRN depletion by shRNA increased cellular sensitivity to Cr(VI) by a cell viability assay [11], and confirmed this result here using the more rigorous clonogenic assay. The colony numbers from all Cr(VI) concentrations in the WRN deficient cells were significantly lower than the control cell line (Figure 4A). Telomerase can compensate for WRN roles in protection against telomere loss, replicative senescence and chromosome aberrations [35]. Similarly, we observed that ecotopic expression of telomerase dramatically reduced the sensitivity of WS cells to Cr(VI) toxicity (Figure 4B). This suggests that telomerase can at least partially compensate for WRN roles in protection against Cr(VI) toxicity.

Upon Cr(VI) exposure WRN re-localizes from the nucleoli into nucleoplasmic foci in S-phase cells that co-localized with γH2AX foci [11]. Next, we investigated whether WRN localizes to telomeres in response to Cr(VI)-induced DNA damage using a telomerase negative U2OS cell line that stably expresses an EYFP-WRN fusion protein [15]. EYFP-WRN responds similarly to Cr(VI) treatment as endogenous WRN [11]. We evaluated telomere colocalized WRN foci (TWFs) by two criteria: (1) the average colocalized foci number per cell and (2) the percent of cells showing colocalization. Cr(VI) exposure induced a concentration-dependent increase in both WRN-telomere colocalization criteria (Figure 4D). At 4 µM Cr(VI) the colocalization induction was significantly higher at 48 h exposure (15-fold) compared to 24 h exposure (5-fold), indicating that WRN response to Cr(VI)-induced damage at telomeres is both concentration and time dependent.

### WRN functions in recovery from Cr(VI)-induced telomere damage

**Figure 5:**
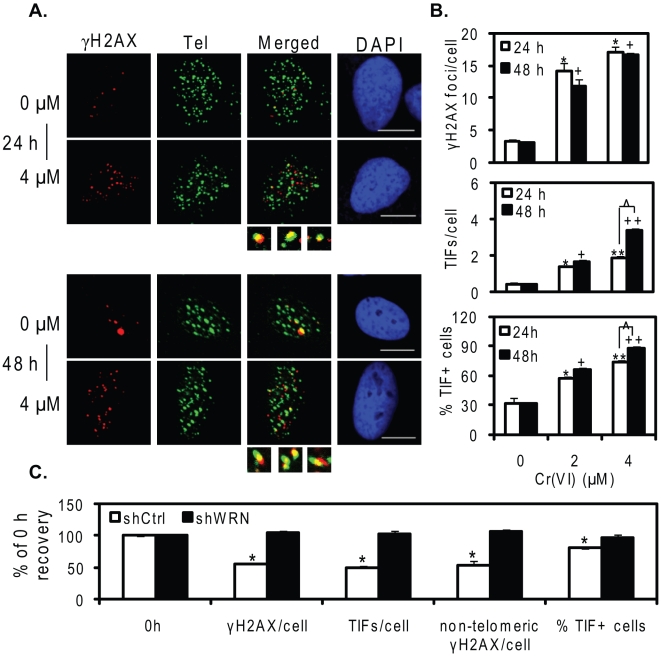
WRN deficiency delays recovery from Cr(VI)-induced telomere damage. (A) Confocal images of EYFP-WRN U2OS cells exposed to the indicated Cr(VI) concentrations for 24 or 48 h. Cells were subjected to IF-FISH analysis of γH2AX (red) and telomere (green) colocalization (yellow). (B) Average γH2AX foci and TIF number per cell and the percent of TIF positive cells. Bars with a different number of symbols are significantly different from each other within each treatment time point (p<0.05). (C) Recovery kinetics of DNA damage and TIFs following Cr(VI) treatment. Cells were exposed to 4 µM Cr(VI) for 48 h and then cultured in Cr(VI)-free medium for 12 h. The values at 12 h recovery were expressed as percent of the values at 0 hr recovery (immediately after the 48 h Cr(VI) treatment). The average number of γH2AX foci, TIFs and non-telomeric γH2AX foci per cell was calculated along with the percent of TIF positive cells. The data represent mean ±SE from two independent experiments, based on at least 50 randomly chosen cells for each Cr(VI) treatment. Symbol(s) above a bar indicates significantly different from the values at 4 µM Cr(VI) treatment for 48 h (p<0.05).

Next, we asked whether the pattern of WRN foci localization is associated with Cr(VI)-induced telomere damage, as indicated by TIFs. The Cr(VI) cellular exposure experiment in Figure 4 also induced a concentration-dependent increase in γH2AX foci and TIF formation (Figure 5B), as observed for WI38 cells. In addition, a significant difference was observed between 24 and 48 h at 4 µM Cr(VI) with regard to TIF formation, but not γH2AX formation, suggesting increased telomere specific damage upon Cr(VI) treatment for a longer time. A time dependent increase was also observed for WRN co-localization to telomeres after 4 µM Cr(VI) exposure (Figure 4C), indicating the pattern of Cr(VI) induced TIF formation and WRN localization is similar (Figures 4C and 5B).

To test whether WRN functions in repair of Cr(VI)-induced telomere damage, we employed U2OS cell lines that stably express either a short hairpin (sh) RNA targeting WRN mRNA or a control shRNA [11]. These two cell lines exhibited similar levels of γH2AX and TIFs immediately after Cr(VI) exposure (Figure S1). However, we previously observed that WRN deficiency results in slower reduction of γH2AX foci during recovery from Cr(VI) [11]. We asked whether this occurred with respect to Cr(VI)-induced TIFs. Cells were exposed to 4 µM Cr(VI) for 48 h, then cultured in Cr(VI)-free medium for 12 h, and then assayed for the average γH2AX foci, TIFs, and non-telomeric γH2AX foci per cell, as well as the percent TIF positive cells, compared to 0 h recovery. Following the recovery in Cr(VI)-free medium, all parameters showed a significant reduction in the shCtrl cells (Figure 5C). Interestingly, the reduction in telomeric γH2AX and non-telomeric γH2AX was similar, indicating no particular bias for repair of telomeric vs. non-telomerc damage. In sharp contrast, the WRN depleted cells did not exhibit a significant reduction in Cr(VI) induced telomeric or non-telomeric damage after recovery. These data strongly suggest that WRN does not prevent Cr(VI)-induced stalled replication forks or DSBs at telomeric or non-telomeric sites, but acts to repair Cr(VI)-induced damage, most likely damaged replication forks, during recovery.

### WRN protects against Cr(VI) induced telomeric defects

Based on the evidence that WRN depleted cells exhibit reduced recovery from telomere damage (Figures 4-5), we asked if WRN prevents Cr(VI) induced telomeric defects. For this purpose, telomeres were stained on chromosome metaphase spreads to visualize telomeric defects in WRN deficient cells compared to controls. We first attempted to knock down WRN expression in WI38 lung cells, however, using two strategies for shWRN expression via retrovirus or lentivirus [36], [37] we were unable to recover viable cells. Furthermore, attempts to recover metaphase spreads after Cr(VI) exposure of WS cells was unsuccessful, although untreated WS cells did yield metaphase spreads. This is likely due to WRN roles in recovery from Cr(VI)-induced replicative stress. As an alternative strategy, we used the shCtrl and shWRN U2OS cell lines in this experiment. U2OS cells are telomerase negative and use the alternative lengthening telomere (ALT) pathway to maintain telomere status [38]. Importantly, WRN depletion in U2OS cells does not impair proliferation [37], but does increase sensitivity to Cr(VI) toxicity (Figure 4).

**Figure 6:**
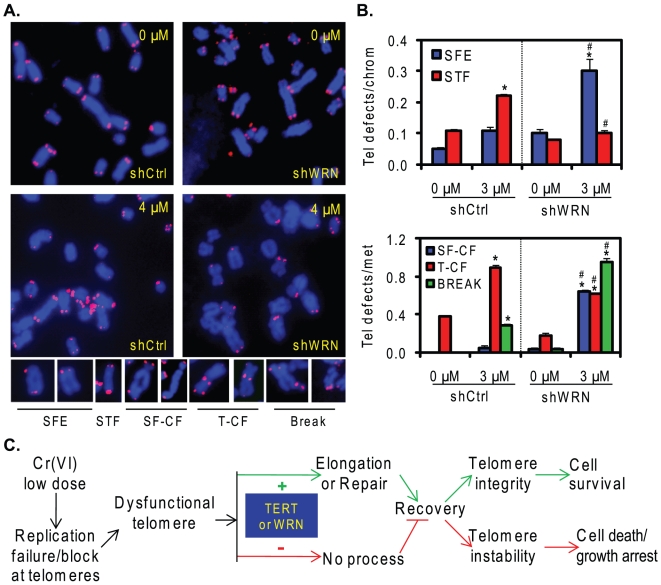
WRN protects against Cr(VI) induced telomere loss. (A) Representative telomere FISH of partial metaphases and telomere defects from shCtrl and shWRN cells exposed to 0 and 3 µM Cr(VI) for 48 h and cultured in Cr(VI)-free medium for 10 h. (B) Cr(VI)-induced telomere instability. Approximately 40 metaphases from two independent experiments were analyzed to quantitate Cr(VI)-induced telomere defects. Bars with a symbol of * indicates a significant difference between 0 and 3 µM in the same cell line and bars with a symbol of # indicates significant difference between exposed shCtrl and shWRN cells (p<0.05). Telomere signal free ends, SFE; telomere sister chromatid fusions, SFT; chromosome/chromatid fusions lacking a telomere signal, SF-CF; chromosome/chromatid fusion with telomere signal at the fusion, T-CF; break indicates chromatid break. (C) Model for roles of WRN and telomerase in protection against Cr(VI)-induced telomere damage.

To directly test Cr(VI)-induced telomeric defects in WRN depleted cells, compared to WRN proficient cells, the shWRN and shCtrl U2OS cell lines were exposed to 3 µM Cr(VI) for 48 h and recovered in Cr(VI)-free media with colcemid for 10 h. We observed several types of telomeric defects [39] (Figure 6A, Figure S2), and compared exposed to unexposed cells (*  =  significant difference) within the same cell line, as well as exposed shWRN to exposed shCtrl cells (#  =  significant difference). After Cr(VI) exposure the incidence of telomere loss (signal-free ends, SFEs) increased in both shCtrl and shWRN cells, compared to untreated cells, but the levels of SFEs was significantly higher in shWRN cells compared to exposed shCtrl cells (#) (Figure 6B). In contrast, this pattern was reversed with respect to sister telomere fusions (STF), since Cr(VI)-induced STFs was significant in shCtrl cells compared with a slight but non-significant induction in shWRN cells (Figure 6B). Consistent with the significant increase in telomere loss (SFEs) in exposed shWRN cells, chromosome/chromatid fusions that lacked telomere signal at the fusion point (SF-CFs) were also significantly increased in exposed shWRN cells compared to untreated shWRN cells (*) and exposed shCtrl cells (#) (Figure 6B). However, while Cr(VI) exposure induced a significant increase in chromosome/chromatid fusions that retain telomere sequence at the fusion point (T-CF) in both cells lines, the T-CF levels in exposed shCtrl cells were significantly higher than in exposed shWRN cells (#) (Figure 6B). Together these data support a role for WRN in preventing Cr(VI) induced telomere loss on sister chromatids that can lead to chromosome or chromatid end fusions that lack a telomere signal. However, WRN was not effective in preventing Cr(VI) induction of chromatid and chromosome fusions that retained telomere staining at the point of fusion.

Recent reports indicate that WRN prevents the induction of chromatid breaks at fragile sites due to replication fork stalling [40], and Cr(VI) exposure is known to induce chromatid breaks [21]. An average of 0.28 spontaneous chromatid breaks per metaphase was observed in shWRN cells, but not in control cells (Figure 6B). Cr(VI) exposure induced a significant increase in chromatid breaks in both cell lines, however, the break incidence in exposed shWRN cells was significantly higher than in exposed shCtrl cells (3.4-fold) (Figure 6B). In summary, our these data support a role for WRN in the protection against Cr(VI) induced telomere loss and chromatid breaks.

## Discussion

Telomere instability is linked to human diseases and cancer development, and several reports indicate that telomeres are vulnerable to oxidative and replication stress. Cr(VI) is an environmental lung carcinogen that induces lesions that interfere with DNA replication. In this study, we show that Cr(VI) exposure leads to telomere damage and chromosomal telomere loss and aberrations associated with Cr(VI)-induced replication stress. Telomerase expression alleviates Cr(VI)-induced telomere instability, and may explain the hypersensitive phenotype of telomerase negative cells to Cr(VI) toxicity. WRN protein promotes replication fork recovery and telomere replication [37], and we found that WRN depletion leads to Cr(VI) hypersensitivity that can be rescued by telomerase. Furthermore, WRN localizes to Cr(VI) induced damaged telomere foci where it promotes their reduction and repair during recovery in Cr(VI)-free media. WRN depletion increased the incidence of chromosomal telomere loss and chromatid breaks induced by Cr(VI) exposure, consistent with roles for WRN in promoting telomere preservation after replication stress. Collectively, our data suggest that environmentally induced replication stress by DNA damaging agents that target G runs can promote telomere instability and aberrations.

Telomerase confers cellular resistance to genotoxins with different modes of action [41]. Similar to previous reports, we found that telomerase expression decreased sensitivity to Cr(VI)-induced toxicity in BJ foreskin fibroblasts [19], [20] (Figure 1). The protective effect of telomerase was associated with significantly lowered apoptosis, senescence, and genomic instability [19]. Surprisingly, the mean telomere lengths were not significantly altered [19], which suggested that telomerase may confer resistance by inhibiting apoptosis or a bystander effect that induces DNA damage in neighboring cells [42]. However, these studies used STELA to measure the distribution of individual telomere lengths which relies on an intact telomeric 3′ tail [19], [43]. WS cells also did not exhibit differences in telomere length by STELA [44], but showed an increase in telomere loss on sister chromatids by telo-FISH that was rescued by telomerase [14], [35]. Similarly, we observed that Cr(VI) significantly increased chromosomal telomere loss, indicated by telomere signal free ends, and sister telomere fusions that were rescued by telomerase (Figure 1B and C). These telomere chromosomal aberrations compromise the telomere 3′tail and thus, cannot be detected by STELA. However, the same study that examined telomere length by STELA also found that Cr(VI) induced dicentric chromosomes and nucleoplasmic bridges in the hTERT- BJ cells [19], which can result from the fusion of dysfunctional telomeres [35]. Therefore, our findings that Cr(VI) exposure induces telomere loss and telomere fusions in both skin and lung telomerase negative cell lines (Figures 1 and 3) are consistent with their results. It is worth noting that a previous study found telomerase did not alter the sensitivity of bronchial fibroblasts to particulate Cr(VI) [45], which may be due to differences in particulate versus soluble chromium and cell lines. Never-the-less, our data provide novel evidence that Cr(VI) exposure induces telomere aberrations than can be rescued by telomerase.

Together with previous work our findings support a role for Cr(VI)-induced replication stress in generating telomeric defects, rather than oxidative stress which can be induced by genotoxic metals and can target telomeres for damage. Guanine is particularly susceptible to oxidative damage and chronic oxidative stress accelerates telomere attrition [46]. Environmental metals can induce reactive oxygen species (ROS), and oxidative stress contributes to arsenic induced apoptosis, accelerated telomere shortening and chromosome aberrations, which are attenuated by telomerase activity [47], [48]. However, Cr(VI) exerts its genotoxic effects through different mechanisms. Although Cr(VI) at higher concentrations can induce ROS, the mutagenic and genotoxic effects at occupationally relevant levels are ascribed to various Cr-DNA and Cr-protein-DNA adducts that impede replication fork progression particularly at G-runs as in telomere repeats [11], [24], [25], [28]. Together with these reports, our results strongly support roles for replicative stress in Cr(VI) induced telomere instability, rather than oxidative stress. First, Cr(VI) exposure results in the accumulation of cells in S-phase [11], [24], [49], and γH2AX foci formation in S-phase cells (Figure 2E). These results indicate that the Cr(VI) induced γH2AX foci at telomeres (TIFs) we observed primarily occurred in S-phase cells (Figure 2C), consistent with replication fork stalling at telomeres. Second, WRN protein localizes to telomeres upon Cr(VI) exposure associated with telomere damage (Figures 4C and 5). Similarly, WRN is recruited to telomeres in S-phase in response to a G-quadruplex stabilizing agent that interferes with telomere replication [7]. Third, Cr(VI) exposure leads to various types of chromosomal telomeric defects in both skin and lung primary fibroblasts (Figures 1 and 3), that were also induced with the G-quadruplex stabilizing agents [7], [50]. Finally, our experiments were conducted at 5% O_2_ to mimic physiological conditions and minimize oxidative stress induced by culture conditions, and while oxidative stress causes accelerated shortening of mean telomere lengths [47], Cr(VI) exposure does not [19]. These results indicate that environmentally induced DNA replicative stress can induce telomeric aberrations.

WRN is implicated in telomere preservation by resolving alternate structures at telomeres during replication, and by restoring stalled or broken replication forks [10], [33]. Our data indicate that WRN responds to Cr(VI) induced telomere damage. The hypersensitivity of WRN deficient cells to Cr(VI) toxicity is greatly reduced by telomerase expression (Figure 4), and Cr(VI) induced WRN localization to telomeres correlates well with telomere damage foci (Figure 4B). However, we found WRN does not prevent telomere damage or stalled forks indicated by γH2AX foci or TIF formation upon Cr(VI) exposure (Figure S1). This is consistent with reports that WRN does not stabilize the disrupted replication forks upon replication stress, but rather facilitates recovery of the replication forks [11], [51], [52]. WRN depletion delayed significantly the reduction in telomeric and non-telomeric γH2AX foci during recovery in Cr(VI) free media (Figure 5C), indicating that WRN likely functions in the repair of stalled or broken replication forks at genomic and telomeric loci.

WRN prevents stochastic telomere loss presumably by facilitating telomere replication and resolving alternate structures [14]. We now report that WRN is also important for preventing telomere loss induced by environmental sources of replication stress. Cr(VI) exposure led to a significant increase in chromosomal ends and fusions that lack telomere signals in WRN deficient U2OS cells, compared to WRN proficient cells (Figure 6). Telomere dysfunction leads to repair by non-homologous end joining or homologous recombination that can induce chromosome or chromatid fusions [8], [53]. When chromosome fusions are caused by telomere deprotection, such as loss of shelterin proteins, the TTAGGG repeats are maintained and appear at the fusion sites [8], [54]. However, the increased Cr(VI) induced fusions in WRN depleted cells lacked detectable TTAGGG repeats at the fusion sites, suggesting the fusions resulted from telomere loss. Consistent with this, WS fibroblasts in which p53 and Rb were inhibited exhibit spontaneous chromosome fusions and anaphase bridges that lack telomere repeats at the fusion points, and that are prevented by telomerase [35]. Telomerase-mediated elongation of critically short or missing telomere ends may compensate for WRN, and rescue the hypersensitivity of WS cells to Cr(VI). In contrast, WRN depletion in U2OS cells led to a reduction in Cr(VI) induced chromosome and chromatid fusions that retain detectable telomere sequence at the fusion site (Figure 6), and presumably resulted from telomere deprotection due to shelterin protein dysfunction rather than telomere loss. Previous reports indicate that in the absence of proper regulation by shelterin proteins, the WRN homolog in yeast can promote aberrant processing, chromosome fusion and telomere loss [55], [56]. For example, telomeric POT1 inhibits WRN exonuclease digestion of telomeric 3′overhangs [57], [58]. Inappropriate processing by WRN can be detrimental since a recent report found WRN induces slow growth of top3 mutant yeast strains [59]. Our study provides the first molecular evidence that WRN protects against environmentally induced telomere loss and downstream chromosome/chromatid fusions associated with replication stress.

In summary, our data show that environmentally induced replication stress can lead to telomeric aberrations and instability that are attenuated by telomerase expression or WRN protein activity. Furthermore, our data suggest that telomeric aberrations contribute to Cr(VI) induced cytotoxcitiy and genotoxicity, and may contribute to respiratory cancers resulting from Cr(VI) exposure. Thus, we provide novel evidence that an environmental pollutant can induce telomere instability, which may contribute to environmentally relevant diseases including cancer.

## Materials and Methods

### Cell lines and culture conditions

Werner syndrome (WS) skin fibroblasts (AG03141) and WI-38 lung fibroblasts were from the Coriell Institute (Camden, NJ). The telomerase-immortalized WS cell line (AG03141) was a gift from Dr. Junko Oshima (University of Washington). BJ and telomerase-immortalized BJ (hTERT BJ) skin fibroblasts were kindly provided by Dr. Peter Lansdorp (University of British Columbia). Cells were cultured in Dulbecco's Modified Eagle Media (Invitrogen, Carlsbad, CA) supplemented with 15% fetal bovine serum, penicillin (50 units/ml), and streptomycin (50 µg/ml) in humidified chambers with 5% CO_2_ and 5% O_2_ at 37°C. Human U2OS osteosarcoma cell line (ATCC) was cultured similarly except with 10% FBS [11], [15]. U2OS cell lines stably expressing a short hairpin RNA against WRN (shWRN) or a scrambled control (shCtrl), or stably expressing WRN with an EYFP fluorescent tag (EYFP-WRN U2OS) were cultured as described previously [11], [15].

### Cellular Cr(VI) exposures

Cells were exposed to potassium dichromate (K_2_Cr_2_O_7_; Cr(VI) (Sigma-Aldrich, St. Louis, MO) as described previously [11], for either 24 or 48 hr at various concentrations as indicated in the figure legends. Some experiments included recovery in Cr (VI)-free media as described in the figure legends. WI-38 cells were irradiated with 1 Gy of γ-irradiation using a Shepherd model 143-45A irradiator (J. L. Shepherd & Associates, CA) followed by 1 h recovery as a negative control for DSB formation [60].

### Cell survival assays

The cell viability assay (CVA) was conducted as previously described with slight modification [11]. Following Cr(VI) exposure 4×10^4^ cells were subcultured in 10-cm culture dish for 7 days. In the clonogenic assay, different cell numbers (800–35,000) depending on Cr(VI) concentrations, were seeded in 6-cm culture dishes and incubated overnight. After Cr(VI) exposure, the cells were cultured in Cr(VI)-free medium for 7 days. Then cells were stained (50% methanol, 7% acetic acid, 0.1% Comassie brilliant blue) for 15 min, and colonies composed of 25 or more cells were counted. The survival fraction at each Cr(VI) concentration was determined by dividing the average number of colonies on treated plates by the average number of colonies on untreated plates after adjusting for the initial seeding cell number (plating factor). Each Cr(VI) concentration exposure was performed in triplicate for each of four independent experiments.

### Immunofluorescence

The association of γH2AX with S-phase cells was detected by double immunostaining with antibodies against γH2AX and incorporated BrdU as previously described with slight modification [11]. WI-38 cells were exposed to various concentrations of Cr(VI) then subjected to 10 µM BrdU pulse-label for 30 min [40], followed by double immunostaining.

### Immunofluorescene-Fluoresence In Situ Hybridization (IF-FISH)

The IF-FISH assay was performed as described previously with modification [2]. Immediately following Cr(VI) exposures or after a 12 h recovery period in Cr(VI)-free media as described in the figure legends, the cells were fixed with 2% paraformaldehyde for 15 min followed by permeabilization and blocking (1 mg/ml BSA, 3% FBS serum, 0.1% Triton X-100, 1 mM EDTA [pH 8.0] in PBS) for 1 h. Then cells were immuno-stained with mouse anti-γH2AX monoclonal antibody (1∶500; Upstate, Billerica, MA) or rabbit anti-GFP polyclonal antibody (1∶400; GeneTex, Irvine, CA) [37]. Next, cells were incubated with either Cy5-conjugated goat anti-mouse (JIR laboratories, Inc., 1∶400) or anti-rabbit (JIR laboratories, Inc., 1∶400) or Alexa 488-conjugated (Invitrogen, 1∶1000) goat anti-mouse secondary antibody, followed by fixation in 2% paraformaldehyde for 5 min. Samples were dehydrated in 70%, 95%, 100% ethanol (5 min each) and then denatured for 10 min at 80°C in hybridization solution (70% deionized formamide, 10% NEN blocking reagent [Roche], 0.1 M Tris-HCl [pH 7.4], MgCl_2_ buffer [82 mM NaH_2_PO_4_, 9 mM citric acid, 20 mM MgCl_2_], and 0.5 µg/ml Cy3-OO-(CCCTAA)_3_ PNA probe (Panagene, South Korea)). After 2 h hybridization at room temperature, the samples were washed twice with wash solution (70% deionized formamide and 10 mM Tris-HCl [pH 7.4]). Samples were counterstained with DAPI, mounted onto slides and images were acquired with an Olympus FluoView 1000 confocal microscope (Olympus America, Inc., NY) as described previously [11].

### Chromsomal Telomere Fluorescent In Situ Hybridization (Telo-FISH)

WI-38 (1.0×10^5^), BJ (2.0×10^5^), hTERT BJ (6.0×10^4^), shWRN U2OS (1.0×10^5^) or shCtrl U2OS (1.0×10^5^) cells were seeded in 10-cm culture dishes and incubated for 2 days. After Cr(VI) exposures, the cells were treated with 0.05 µg/ml colcemid (Invitrogen) for 10 h. Telomere FISH on metaphase chromosomes was performed as described previously with some modification [31]. Cells were harvested and treated with 75 mM KCl hypotonic buffer for 12 min at 37°C and then fixed and stored in methanol/acetic acid fixative (3∶1). Cells were dropped onto slides and aged overnight. Next, cells were fixed in 4% formaldehyde in PBS for 2 min, washed with PBS and treated with 0.1% pepsin in 0.01 N HCl for 10 min at 37°C. Fixation and washing were repeated. Subsequently, slides were dehydrated in an ethanol series of 70, 90 and 100% for 5 min and air-dried. Then samples were denatured for 3 min at 80°C in the same hybridization solution as in the IF-FISH. After 2 h hybridization at room temperature, the slides were washed twice for 20 min each with wash solution I (70% deionized formamide, 10 mM Tris-HCl [pH 7.4], and 0.01% BSA) and three times 15 min each with wash solution II (100 mM Tris-HCl [pH 7.4], 66.7 mM NaCl, and 0.1% Tween 20). The samples were counterstained with DAPI and mounted with coverslips.

The images of metaphases were obtained with Nikon Ti90 epi-fluorescence microscope (Nikon Inc., NY) equipped with PlanApo 60×/1.40 oil immersion objective. The NIS element advanced software was used to acquire and analyze the images with the same settings for paired cell lines in each experiment. In order to rigorously identify and qualify telomere staining and telomere signal free chromosome ends, fusions and aberrations, a series of z-stacked images (0.15 µm steps) were acquired for each metaphase and analyzed. This technique allowed for rigorous distinction of a telomere signal that was lost from a telomere signal that was out of focus.

### Statistical methods

All statistical analyses were conducted with SAS software (SAS, Version 9.2, NC). Student t-test was used to determine the significance of differences between two treatments or time points. To determine the significance of differences among more than two treatments or time points, one-way ANOVA followed by Duncan's multiple comparison test was employed. The statistically significant level was set at p<0.05.

## Supporting Information

Table S1 Percent telomere defects (number of defects/number of chromosomes). The cells were exposed to the indicated Cr(VI) doses for 48 h and cultured in Cr(VI)-free medium for 10 h.
(0.70 MB EPS)

Figure S1 WRN does not prevent TIF formation. Confocal images of shCtrl and shWRN U2OS cells exposed to 4 μM Cr(VI) for 48 h (A) and then cultured in Cr(VI)-free medium for 12 h (B). (C) Average γH2AX foci and TIF number per cell and the percent of TIF positive cells from (A). The data represent mean ±SE from two independent experiments, based on at least 50 randomly chosen cells for each Cr(VI) treatment.
(1.96 MB EPS)

Figure S2 WRN deficiency does not affect Cr(VI)-induced doublets and telomeric DNA-containing double minute chromosomes (TDMs). shCtrl and shWRN cells were exposed to 0 and 3 μM Cr(VI) for 48 h and cultured in Cr(VI)-free medium for 10 h. (A) Average doublets per chromosome. (B) Average TDMs per chromosome. Around 40 metaphases from two independent experiments were analyzed to quantitate Cr(VI)-induced telomere instability.
(0.70 MB EPS)

## References

[1] Palm W, de Lange T (2008). How shelterin protects mammalian telomeres.. Annu Rev Genet.

[2] Takai H, Smogorzewska A, de Lange T (2003). DNA damage foci at dysfunctional telomeres.. Curr Biol.

[3] d'Adda di Fagagna F, Reaper PM, Clay-Farrace L, Fiegler H, Carr P (2003). A DNA damage checkpoint response in telomere-initiated senescence.. Nature.

[4] Bodnar AG, Ouellette M, Frolkis M, Holt SE, Chiu CP (1998). Extension of life-span by introduction of telomerase into normal human cells.. Science.

[5] Harley CB, Futcher AB, Greider CW (1990). Telomeres shorten during ageing of human fibroblasts.. Nature.

[6] Calado RT, Young NS (2009). Telomere diseases.. N Engl J Med.

[7] Rizzo A, Salvati E, Porru M, D'Angelo C, Stevens MF (2009). Stabilization of quadruplex DNA perturbs telomere replication leading to the activation of an ATR-dependent ATM signaling pathway.. Nucleic Acids Res.

[8] Smogorzewska A, Karlseder J, Holtgreve-Grez H, Jauch A, de Lange T (2002). DNA ligase IV-dependent NHEJ of deprotected mammalian telomeres in G1 and G2.. Curr Biol.

[9] Arnoult N, Saintome C, Ourliac-Garnier I, Riou JF, Londono-Vallejo A (2009). Human POT1 is required for efficient telomere C-rich strand replication in the absence of WRN.. Genes Dev.

[10] Rossi ML, Ghosh AK, Bohr VA Roles of Werner syndrome protein in protection of genome integrity..

[11] Liu FJ, Barchowsky A, Opresko PL (2009). The Werner syndrome protein functions in repair of Cr(VI)-induced replication-associated DNA damage.. Toxicol Sci.

[12] Zecevic A, Menard H, Gurel V, Hagan E, DeCaro R (2009). WRN helicase promotes repair of DNA double-strand breaks caused by aberrant mismatch repair of chromium-DNA adducts.. Cell Cycle.

[13] Sidorova JM, Li N, Folch A, Monnat RJ (2008). The RecQ helicase WRN is required for normal replication fork progression after DNA damage or replication fork arrest.. Cell Cycle.

[14] Crabbe L, Verdun RE, Haggblom CI, Karlseder J (2004). Defective telomere lagging strand synthesis in cells lacking WRN helicase activity.. Science.

[15] Opresko PL, Otterlei M, Graakjaer J, Bruheim P, Dawut L (2004). The Werner syndrome helicase and exonuclease cooperate to resolve telomeric D loops in a manner regulated by TRF1 and TRF2.. Mol Cell.

[16] Opresko PL, Fan J, Danzy S, Wilson DM, Bohr VA (2005). Oxidative damage in telomeric DNA disrupts recognition by TRF1 and TRF2.. Nucleic Acids Res.

[17] P.H.S (2000).

[18] Wu X, Amos CI, Zhu Y, Zhao H, Grossman BH (2003). Telomere dysfunction: a potential cancer predisposition factor.. J Natl Cancer Inst.

[19] Glaviano A, Nayak V, Cabuy E, Baird DM, Yin Z (2006). Effects of hTERT on metal ion-induced genomic instability.. Oncogene.

[20] Glaviano A, Mothersill C, Case CP, Rubio MA, Newson R (2009). Effects of hTERT on genomic instability caused by either metal or radiation or combined exposure.. Mutagenesis.

[21] Wise SS, Holmes AL, Wise JP (2008). Hexavalent chromium-induced DNA damage and repair mechanisms.. Rev Environ Health.

[22] Quievryn G, Peterson E, Messer J, Zhitkovich A (2003). Genotoxicity and mutagenicity of chromium(VI)/ascorbate-generated DNA adducts in human and bacterial cells.. Biochemistry.

[23] Slade PG, Hailer MK, Martin BD, Sugden KD (2005). Guanine-specific oxidation of double-stranded DNA by Cr(VI) and ascorbic acid forms spiroiminodihydantoin and 8-oxo-2′-deoxyguanosine.. Chem Res Toxicol.

[24] Xu J, Bubley GJ, Detrick B, Blankenship LJ, Patierno SR (1996). Chromium(VI) treatment of normal human lung cells results in guanine-specific DNA polymerase arrest, DNA-DNA cross-links and S-phase blockade of cell cycle.. Carcinogenesis.

[25] Bridgewater LC, Manning FC, Patierno SR (1998). Arrest of replication by mammalian DNA polymerases alpha and beta caused by chromium-DNA lesions.. Mol Carcinog.

[26] Ha L, Ceryak S, Patierno SR (2004). Generation of S phase-dependent DNA double-strand breaks by Cr(VI) exposure: involvement of ATM in Cr(VI) induction of gamma-H2AX.. Carcinogenesis.

[27] Xie H, Holmes AL, Young JL, Qin Q, Joyce K (2009). Zinc chromate induces chromosome instability and DNA double strand breaks in human lung cells.. Toxicol Appl Pharmacol.

[28] Tamblyn L, Li E, Sarras H, Srikanth P, Hande MP (2009). A role for Mus81 in the repair of chromium-induced DNA damage.. Mutat Res.

[29] Stackpole MM, Wise SS, Goodale BC, Duzevik EG, Munroe RC (2007). Homologous recombination repair protects against particulate chromate-induced chromosome instability in Chinese hamster cells.. Mutat Res.

[30] Bryant HE, Ying S, Helleday T (2006). Homologous recombination is involved in repair of chromium-induced DNA damage in mammalian cells.. Mutat Res.

[31] Poon SS, Lansdorp PM (2001). Quantitative fluorescence in situ hybridization(Q-FISH).. Curr Protoc Cell Biol Chapter.

[32] Davalos AR, Campisi J (2003). Bloom syndrome cells undergo p53-dependent apoptosis and delayed assembly of BRCA1 and NBS1 repair complexes at stalled replication forks.. J Cell Biol.

[33] Opresko PL (2008). Telomere ResQue and preservation—roles for the Werner syndrome protein and other RecQ helicases.. Mech Ageing Dev.

[34] Multani AS, Chang S (2007). WRN at telomeres: implications for aging and cancer.. J Cell Sci.

[35] Crabbe L, Jauch A, Naeger CM, Holtgreve-Grez H, Karlseder J (2007). Telomere dysfunction as a cause of genomic instability in Werner syndrome.. Proc Natl Acad Sci U S A.

[36] Grandori C, Wu KJ, Fernandez P, Ngouenet C, Grim J (2003). Werner syndrome protein limits MYC-induced cellular senescence.. Genes Dev.

[37] Opresko PL, Calvo JP, von Kobbe C (2007). Role for the Werner syndrome protein in the promotion of tumor cell growth.. Mech Ageing Dev.

[38] Gagos S, Chiourea M, Christodoulidou A, Apostolou E, Raftopoulou C (2008). Pericentromeric instability and spontaneous emergence of human neoacrocentric and minute chromosomes in the alternative pathway of telomere lengthening.. Cancer Res.

[39] Zhu XD, Niedernhofer L, Kuster B, Mann M, Hoeijmakers JH (2003). ERCC1/XPF removes the 3′ overhang from uncapped telomeres and represses formation of telomeric DNA-containing double minute chromosomes.. Mol Cell.

[40] Pirzio LM, Pichierri P, Bignami M, Franchitto A (2008). Werner syndrome helicase activity is essential in maintaining fragile site stability.. J Cell Biol.

[41] Rubio MA, Davalos AR, Campisi J (2004). Telomere length mediates the effects of telomerase on the cellular response to genotoxic stress.. Exp Cell Res.

[42] Cogan N, Baird DM, Phillips R, Crompton LA, Caldwell MA DNA damaging bystander signalling from stem cells, cancer cells and fibroblasts after Cr(VI) exposure and its dependence on telomerase.. Mutat Res.

[43] Baird DM (2005). New developments in telomere length analysis.. Exp Gerontol.

[44] Baird DM, Davis T, Rowson J, Jones CJ, Kipling D (2004). Normal telomere erosion rates at the single cell level in Werner syndrome fibroblast cells.. Hum Mol Genet.

[45] Wise SS, Elmore LW, Holt SE, Little JE, Antonucci PG (2004). Telomerase-mediated lifespan extension of human bronchial cells does not affect hexavalent chromium-induced cytotoxicity or genotoxicity.. Mol Cell Biochem.

[46] von Zglinicki T (2002). Oxidative stress shortens telomeres.. Trends Biochem Sci.

[47] Liu L, Trimarchi JR, Navarro P, Blasco MA, Keefe DL (2003). Oxidative stress contributes to arsenic-induced telomere attrition, chromosome instability, and apoptosis.. J Biol Chem.

[48] Newman JP, Banerjee B, Fang W, Poonepalli A, Balakrishnan L (2008). Short dysfunctional telomeres impair the repair of arsenite-induced oxidative damage in mouse cells.. J Cell Physiol.

[49] Xie H, Wise SS, Holmes AL, Xu B, Wakeman TP (2005). Carcinogenic lead chromate induces DNA double-strand breaks in human lung cells.. Mutat Res.

[50] Sfeir A, Kosiyatrakul ST, Hockemeyer D, MacRae SL, Karlseder J (2009). Mammalian telomeres resemble fragile sites and require TRF1 for efficient replication.. Cell.

[51] Dhillon KK, Sidorova J, Saintigny Y, Poot M, Gollahon K (2007). Functional role of the Werner syndrome RecQ helicase in human fibroblasts.. Aging Cell.

[52] Pichierri P, Franchitto A, Mosesso P, Palitti F (2001). Werner's syndrome protein is required for correct recovery after replication arrest and DNA damage induced in S-phase of cell cycle.. Mol Biol Cell.

[53] Wang RC, Smogorzewska A, de Lange T (2004). Homologous recombination generates T-loop-sized deletions at human telomeres.. Cell.

[54] van Steensel B, Smogorzewska A, de Lange T (1998). TRF2 protects human telomeres from end-to-end fusions.. Cell.

[55] Wang X, Baumann P (2008). Chromosome fusions following telomere loss are mediated by single-strand annealing.. Mol Cell.

[56] Rog O, Miller KM, Ferreira MG, Cooper JP (2009). Sumoylation of RecQ helicase controls the fate of dysfunctional telomeres.. Mol Cell.

[57] Sowd G, Lei M, Opresko PL (2008). Mechanism and substrate specificity of telomeric protein POT1 stimulation of the Werner syndrome helicase.. Nucleic Acids Res.

[58] Li B, Reddy S, Comai L (2009). Sequence-specific processing of telomeric 3′ overhangs by the Werner syndrome protein exonuclease activity.. Aging (Albany NY).

[59] Aggarwal M, Brosh RM (2009). WRN helicase defective in the premature aging disorder Werner syndrome genetically interacts with topoisomerase 3 and restores the top3 slow growth phenotype of sgs1 top3.. Aging (Albany NY).

[60] Balajee AS, Geard CR (2004). Replication protein A and gamma-H2AX foci assembly is triggered by cellular response to DNA double-strand breaks.. Exp Cell Res.

